# Coefficient datasets for high-order, stable, and conservative boundary schemes for central and compact finite differences

**DOI:** 10.1016/j.dib.2019.104086

**Published:** 2019-06-01

**Authors:** P.T. Brady, D. Livescu

**Affiliations:** CCS-2, Los Alamos National Laboratory, Los Alamos, NM 87544, United States

## Abstract

Stable and conservative numerical boundary schemes, for both compact and explicit (central) finite differences require a number of parameters that must be tuned for stability. Values of these coefficients for 4th, 6th, and 8th boundary schemes are given in this article. The stability of the schemes is demonstrated through a series of numerical tests in “High-Order, Stable, and Conservative Boundary Schemes for Central and Compact Finite Differences” Brady and Livescu, 2019. These tests include: a neutrally stable constant coefficient hyperbolic system, a two-dimensional varying coefficient hyperbolic scalar equation and, examining the transport of an inviscid vortex using the compressible Euler equations. The error norms for the variety of tests associated with different the schemes for different grid resolutions and time-step constraints are given in the accompanying databases.

**Table:** Specifications table

Subject area	*Computational physics*
More specific subject area	*Numerical methods*
Type of data	*ASCII-text files and sqlite3 databases*
How data was acquired	*Numerical simulation*
Data format	*analyzed*
Experimental factors	*A large number of numerical simulations were performed*
Experimental features	*Stable numerical schemes located using a gradient ascent approach*
Data source location	*Los Alamos National Laboratory*
Data accessibility	*Data is with the article*
Related research article	*Brady, P. T & Livescu, D.; High-Order, Stable, and Conservative Boundary Schemes for Central and Compact Finite Differences; Computers & Fluids, Vol 183 (2019) pp. 84-101*

**Table:** 

**Value of the data** • With this data, any researcher may easily choose a high-order, stable numerical boundary scheme to couple with their choice of interior scheme. • Researchers may also try out different free parameters to generate their own numerical boundary schemes, which automatically satisfy discrete conservation constraints. • Analysis of these datasets may provide insight into the development of novel stability theories.

## 1 Data

There are two types of data presented in this article. The first type is an ascii representation of the constraints on the coefficients for each numerical boundary scheme. There are six schemes in total. Explicit (central) differencing schemes of 4th, 6th, and 8th order are labeled “E4.txt”, “E6.txt”, and “E8.txt”, respectively. Compact differencing schemes of 4th, 6th, and 8th order are labeled “T4.txt”, “T6.txt”, and “T8.txt”, where the “T” prefix indicates a compact scheme with tri-diagonal structure. The equations are placed in an ascii format because their length can make copy/paste from a pdf document error prone. The variable names in txt files assumes that a stencil of length “t”, approximating the first derivative, at a point “i”, near the left boundary can be written as:

$$\sum_{k=-s_{1}}^{k=s}\beta_{ik}f_{i+k}^{'}=\;\frac{1}{h}\sum_{j=0}^{j=t-1}\alpha_{ij}f_{j}$$

Where s = 0 for the explicit (central) differencing schemes and s = 1 for the tridiagonal compact schemes. For all schemes, $\beta_{i0}=1.$ To ease the processing of the txt files, the coefficients, $\beta_{i,\pm 1}$are represented as “beta_i_p1/m1”. Each numerical boundary scheme is designed to be coupled to the appropriate centered differencing scheme in the interior of the domain.

The second type of data is a sqlite3 database for each of the schemes. Each sqlite database contains 6 tables. The most important table is “nbs”, for which the columns are a unique “id” followed by the free parameters for the scheme. The 5 other tables record the performance of the numerical boundary scheme on a variety of numerical experiments, which will be described in the next section. The full set of coefficients for any numerical boundary scheme can be computed by substituting the free parameters associated with an “id” from the nbs table into the constraints given in the corresponding txt file. As an example, the following python script (requires python 3) prints the coefficients associated with 15th T6 scheme. Note that this assumes the files “T6.db” and “T6.txt” are in the current directory.

**Figure:**
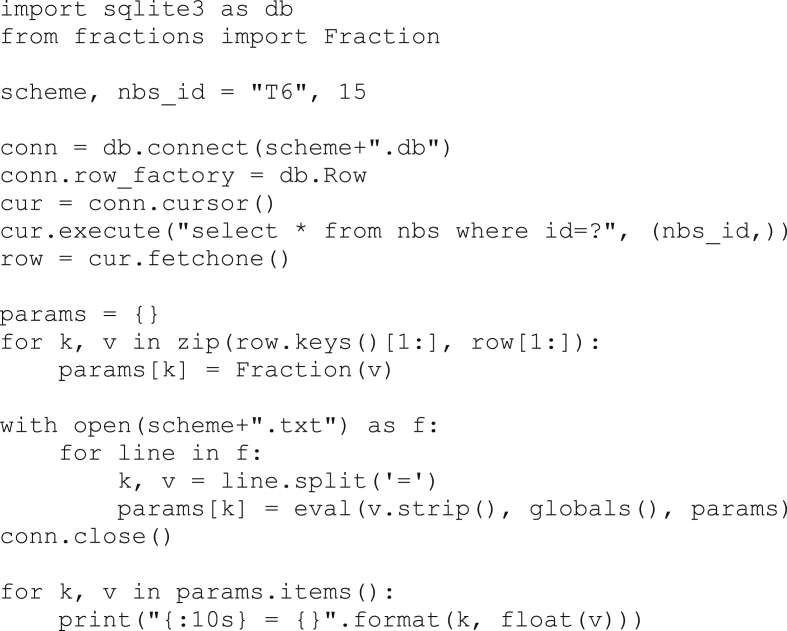

## 2 Experimental design, materials, and methods

The values of the free parameters contained in the “nbs” table were arrived at via the optimization procedure described in [1]. The schemes were subjected to a variety of numerical tests to verify their stability properties. The error norms of these numerical experiments are recorded in the remaining tables in each database. In all numerical tests described below, the spacial discretization is done a uniform Cartesian mesh and time integration is carried out using a standard RK4 method.

The first numerical experiment is recorded in section 4.2 of [1] and is reproduced here for completeness. It consists of solving the hyperbolic system:

$$\frac{\partial u}{\partial t}=\frac{\partial v}{\partial x},\;\;\;\frac{\partial v}{\partial t}=\frac{\partial u}{\partial x},\;\;\;\;\;\;\;\;\;\;\;x\in \left[0,1\right]$$

with boundary and initial conditions:

u(0,t)=v(1,t)=0

$$u\left(x,0\right)=\;-\frac{3\pi }{2}\sin\frac{3\pi x}{2},\;\;\;\;\;\;\;\;\;v\left(x,0\right)=\;0$$

In the table “linear_system_cfl”, the simulations are run to a time of 500 for a large and small CFL at various grid resolutions (denoted by the “nx” column) and the maximum error in u and v are recorded in the “Linf_u” and “Linf_v” columns, respectively.

The table “linear_system_dt” records the same data but with constant timestep simulations so that the order of accuracy of the simulations can be computed. These are recorded in the “order_u” and “order_v” columns.

The second numerical experiment is described in section 4.3 of [1] and is reproduced here for completeness. It consists of solving the two-dimensional, varying coefficient scalar wave equation:

$$\frac{\partial u}{\partial t}+\frac{\partial \varphi }{\partial x}\frac{\partial u}{\partial x}+\frac{\partial \varphi }{\partial y}\frac{\partial u}{\partial y}=0,\;\;\;\;\;\;\;0\leq \left(x,y\right)\leq \sqrt{2}$$

Where $\varphi \left(x,y\right)=\;\sqrt{x{\left(x-0.25\right)}^{2}{+\left(y-0.25\right)}^{2}}$, and with initial and boundary conditions:

u(x,y,0)=sin2πφ

u(0,y,t)=sin2π(φ(0,y)−t)

u(x,0,t)=sin2π(φ(x,0)−t)

In the table “linear_wave2d_cfl”, the simulations are run to a time of 1000 for a large and small CFL at various grid resolutions and the maximum error in u is recorded as “Linf_u”.

The table “linear_wave2d_dt” records the same data but with constant timestep simulations so that the order of accuracy of the simulations can be computed. This is recorded in the “order_u” column.

The final numerical experiment is also described in section 4.4 of [1]. In this test, the two-dimensional Euler equations are solved to examine the transport of vortex through a domain. A vortex of nondimensional circulation, ε=1.5, is placed in the center of a computational domain of length x∈[0,20],y∈[0,10] discretized with $N_{x}N_{y}$ grid points, where $N_{y}=\left(N_{x}+1\right)/2$. The background flow is a uniform flow in the x-direction with a Mach number of 2. The simulations are run to a non-dimensional time of 1000 at various grid resolutions and CFL's. The maximum and final error in pressure is recorded in the “vortex” table in the “Linf_p”, and “Linf_p_final” columns, respectively.

## Appendix A Supplementary data

Multimedia components 1 Multimedia components

Multimedia components 2

Multimedia components 3

Multimedia components 4

Multimedia components 5

Multimedia components 6

Multimedia components 7

Multimedia components 8

Multimedia components 9

Multimedia components 10

Multimedia components 11

Multimedia components 12
